# Hare-Lip Operation

**Published:** 1887-10

**Authors:** 


### Hare-lip Operation.

An unusual case of hare-lip was operated upon by Prof. Lueske. The inter-maxillary bone was not present. The fissure passed back along the median line, where the septum of the nose was also wanting. So flattened was the nose that in profile no trace of it could be seen, what there was of it being entirely concealed by the prominent cheeks. Such cases are exceedingly unpromising, and even after repeated operations must necessarily remain very much deformed.

The infant was bandaged in the lap of an assistant in a sitting posture; in front sat the operator as is his custom. The first step of the operation was to loosen up the alæ of the nose and the neighboring soft parts, in order to render the closing of the very broad fissure possible. The edges were freshened and united in a straight suture line with a great many deep sutures, in order to hold them as securely together as the great tension would allow. No dressing was applied. Several cases of hare-lip have been operated upon in the clinic in the last few weeks and treated afterwards without any probation, usually with good success. In one case the child had a violent crying spell the night after the stitches were removed, and the whole thing tore open again. It is probable that carefully applied strips of adhesive plaster would have prevented the accident.—*Lancet and Clinic.*

COLORLESS TINCTURE OF IODINE.—Iodide of ammonium, ℨij.; Iodoform, ℨss.; Ammonia liquid, ℨss.; Alcohol, ℥iiiss. The mixture should be left exposed to the light eight or ten days, when all trace of color is gone.—*Medical Press.*

THE ANATOMY OF MELANCHOLY.—"There is no doubt," says Sir John Lubbock, "some selfish satisfaction in yielding to melancholy, in brooding over grievances, especially if more or less imaginary, in fancying that we are victims of fate. To be bright and cheerful often requires an effort. There is a certain art in keeping ourselves happy; in this respect as in others we require to watch over and manage ourselves almost as if we were somebody else."—*Medical Press and Circular.*

FIREPROOFING SOLUTION.—For rendering fabrics, wood, and other inflammable objects fireproof, a writer in *La Nature* recommends borotungstate of soda, a salt which he states has never hitherto been employed for the purpose. It is made by dissolving boric acid in a hot solution of tungstate of soda. Objects impregnated with this solution are rendered incombustible. The solution gives off no deleterious gas, while ammoniacal salts, phosphate of ammonia, and salts of phosphorus render the air irrespirable.

